# Hunger and Satiety Mechanisms and Their Potential Exploitation in the Regulation of Food Intake

**DOI:** 10.1007/s13679-015-0184-5

**Published:** 2016-01-14

**Authors:** Tehmina Amin, Julian G. Mercer

**Affiliations:** Rowett Institute of Nutrition and Health, University of Aberdeen, Greenburn Road, Aberdeen, AB21 9SB Scotland UK

**Keywords:** Satiety, Satiation, Hunger, Appetite, Obesity, Food reformulation

## Abstract

Effective strategies to combat recent rises in obesity levels are limited. The accumulation of excess body fat results when energy intake exceeds that expended. Energy balance is controlled by hypothalamic responses, but these can be overridden by hedonic/reward brain systems. This override, combined with unprecedented availability of cheap, energy-dense, palatable foods, may partly explain the increase in overweight and obesity. The complexity of the processes that regulate feeding behaviour has driven the need for further fundamental research. Full4Health is an EU-funded project conceived to advance our understanding of hunger and satiety mechanisms. Food intake has an impact on and is also affected by the gut-brain signalling which controls hunger and appetite. This review describes selected recent research from Full4Health and how new mechanistic findings could be exploited to adapt and control our physiological responses to food, potentially providing an alternative solution to addressing the global problems related to positive energy balance.

## Introduction

The last 30 years has seen an unprecedented rise in global obesity levels; from 1980–2008, worldwide obesity prevalence almost doubled [1]. The growing impact of this trend on health has been profound since obesity is a major risk factor for most non-communicable disease including cardiovascular disease, cancers, chronic respiratory disease and diabetes. This inevitably adds further economic burden on already overstretched healthcare systems [2]. The accumulation of body fat that underlies obesity is fundamentally a reflection of positive energy balance, where energy consumed as food and drink exceeds that expended through metabolism, thermogenesis and physical activity. Nevertheless, the relative stability observed in body weight over extended periods of time for most individuals highlights the existence of a regulatory system of considerable precision given the complexity and patterning of the components that need to be integrated—meals, snacks and drinks of variable energy and macronutrient composition on one side of the equation, and voluntary exercise and the obligatory components of metabolism on the other. Clearly, many of these contributors vary significantly within and between days and over the longer term, and the regulatory system needs to be able to function effectively in the face of this temporal heterogeneity.

Although energy balance has a high degree of precision in its regulation, this regulation is biased in favour of caloric over-consumption to mitigate the risk of starvation, a necessary evolutionary strategy in the past when food supplies were scarce or unpredictable—the ‘feast or famine’ scenario [3]. The system is thus very sensitive to negative energy balance but comparatively tolerant of positive energy balance. Fortunately, famine is now rare in the developed world, where changes in agriculture, production, storage and food processing over the last century have culminated in the plentiful supply of cheap, palatable and nutritious food that we now take for granted. If appetite and feeding behaviour was simply controlled by energy requirement, i.e. if energy balance was the only driver, there would not be an obesity problem. However, it is now increasingly apparent that the hedonic/reward brain systems can override the hypothalamic systems that regulate energy balance and that the variety of energy dense, high sugar and high fat foods that we are now exposed to is hyper-stimulating these systems [4]. This scenario can drive food consumption beyond homeostatic needs, providing a partial explanation for rises in obesity prevalence, most of which can be considered to be diet-induced, but also the outcome of gene-environment interaction [3, 4, 5].

### Strategies for Tackling Food Intake and Obesity

#### Surgical Intervention

It is clear that strategies to combat the growing rise in obesity prevalence are currently limited in number and efficacy. Consequently, one of the most successful interventions in extreme obesity is bariatric surgery [6]. However, the associated risk of complications and mortality, and the potential drain on healthcare budgets, mean that these procedures are not generally considered in less extreme cases of overweight or mild obesity, and it could be argued that this would in any case be inappropriate. Bariatric surgery includes procedures that act by either reducing stomach size or capacity or by bypassing part of the intestine or a combination of the two [7]. Although the surgical restriction in the size of the stomach was initially assumed to be a major factor in resultant weight loss, it has also been shown consistently that a number of procedures result in sustained changes in blood concentrations of gastrointestinal (GI) hormones including those responsible for the incretin effect; glucagon-like peptide-1 (GLP-1) and gastric inhibitory peptide (GIP) [6, 7]. These changes in GI hormone levels are likely to play a role in induction and maintenance of weight loss. Three main surgical procedures are outlined here. Adjustable gastric banding creates a smaller stomach pouch by encircling the stomach with a silicone ring. This purely restrictive method has no effect on the levels of GLP-1, peptide YY (PYY) and GIP [6]. However, sleeve gastrectomy (creates a long, thin stomach by longitudinal stapling), which is also restrictive, increases levels of GLP-1, PYY and GIP and decreases ghrelin levels. Roux-en-Y-gastric bypass (RYGB) is a combination of both stomach restriction and intestinal bypass; a small stomach pouch is created, bypassing the pylorus and duodenum and taking nutrients directly to the ileum. Nutrients do not mix with bile and pancreatic juices until they meet in the newly constructed common limb. Increased levels of GLP-1 and PYY, reductions in GIP, and changes in ghrelin have been consistently observed [6, 7]. Sleeve gastrectomy can result in excess weight loss of 77.5 % BMI at 3 years post-surgery or 53 % BMI at 6 years post-surgery. RYGB can be similarly effective with excess weight loss of 59 % BMI less than 2 years post-surgery and 63 % BMI more than 2 years post-surgery [6]. There is also often rapid resolution of type 2 diabetes post-operatively in obese patients with prior type 2 diabetes, even before weight is lost, which can most likely be attributed to the altered GI hormone levels. The positive outcome of this is long-term weight maintenance by enhanced glucose regulation and appetite reduction [6, 7].

#### Pharmacological Therapy

In the last 20 years, elucidation of the neural pathways controlling hunger, appetite and energy homeostasis, and the feedback of peripheral hormones and metabolites onto these pathways, has provided numerous potential targets for pharmacological intervention. Again, the pharmacological therapies have mostly been targeted to individuals with BMI in excess of 30 for whom their condition is already affecting health and longevity. Despite this, several drugs which made it to market were licensed for only a short time before being withdrawn due to unacceptable side effects [8]. A major reason for limited success in this endeavour is that the gut-brain systems being targeted are complex, involving many feedback mechanisms, and with the target signalling molecules often being distributed in multiple locations. This means that drugs targeting a particular molecule, such as a receptor, in one location and with a relevant function, may have unintended consequences in a different location [4].

This can be illustrated by the example of dexfenfluoramine which increases the bioavailability of 5-hydroxytryptamine (5-HT; serotonin), a neurotransmitter involved in a wide range of functions including energy balance. 5-HT has long been a target in obesity therapy because increasing its levels reduces appetite and hence body weight. Dexfenfluoramine increases availability of 5-HT in all areas, stimulating all 5-HT receptors. Accordingly, significant problems resulted from increased activation of 5-HT in heart valves, leading to valvulopathy, which together with an increased risk of pulmonary hypertension, resulted in dexfenfluoramine being withdrawn from the market [9, 10]. Similarly, rimonabant, a cannabinoid CB1 receptor antagonist, was licensed for use in Europe as an anti-obesity therapy due to its effect in reducing appetite and weight gain. However, it too was subsequently withdrawn from the market due to serious psychiatric side effects including anxiety, depression and suicide [4, 9]. For other potential therapeutics, drug programmes have been shelved or abandoned before making it to market. For example, activation of the melanocortin-4 receptor (MC4R), located in the paraventricular nucleus of the hypothalamus, decreases food intake and increases energy consumption, making it an attractive therapeutic target. Mutations causing MC4R dysfunction result in lack of satiety and reduced energy expenditure leading to severe obesity. However, although a number of MC4R agonists have demonstrated efficacy in preclinical studies, none have progressed beyond phase I or II trials due to undesirable side effects in the clinic, including increased blood pressure and heart rate [11].

#### Food Reformulation and Behaviour Change

The above discussion illustrates that although there may be interventions relevant to individuals with BMI of 30 and above, there is an unmet need for weight management strategies in the wider population where the challenge is to prevent or at least slow the progression into overweight and obesity. Most individuals gain weight slowly over periods of years or decades [3], and we need to find innovative solutions to support weight control in this group. Food is frequently cited, not unreasonably, as being a major part of the problem in weight gain, but an alternative perspective could see the natural properties of particular foods and food components being harnessed to interact with our physiology to naturally limit calorie intake [3]. Can we target components of the satiety cascade (see below; [12]) to promote weight management (weight loss, maintenance of weight loss, restrained weight gain) in the longer term? This approach might not be sufficiently powerful to address preexisting clinical obesity but could support better weight management for the majority of overweight or mildly obese. Such a strategy would be likely to be combined with lifestyle modification, such as exercise [3].

### The Satiety Cascade

**Fig. 1:**
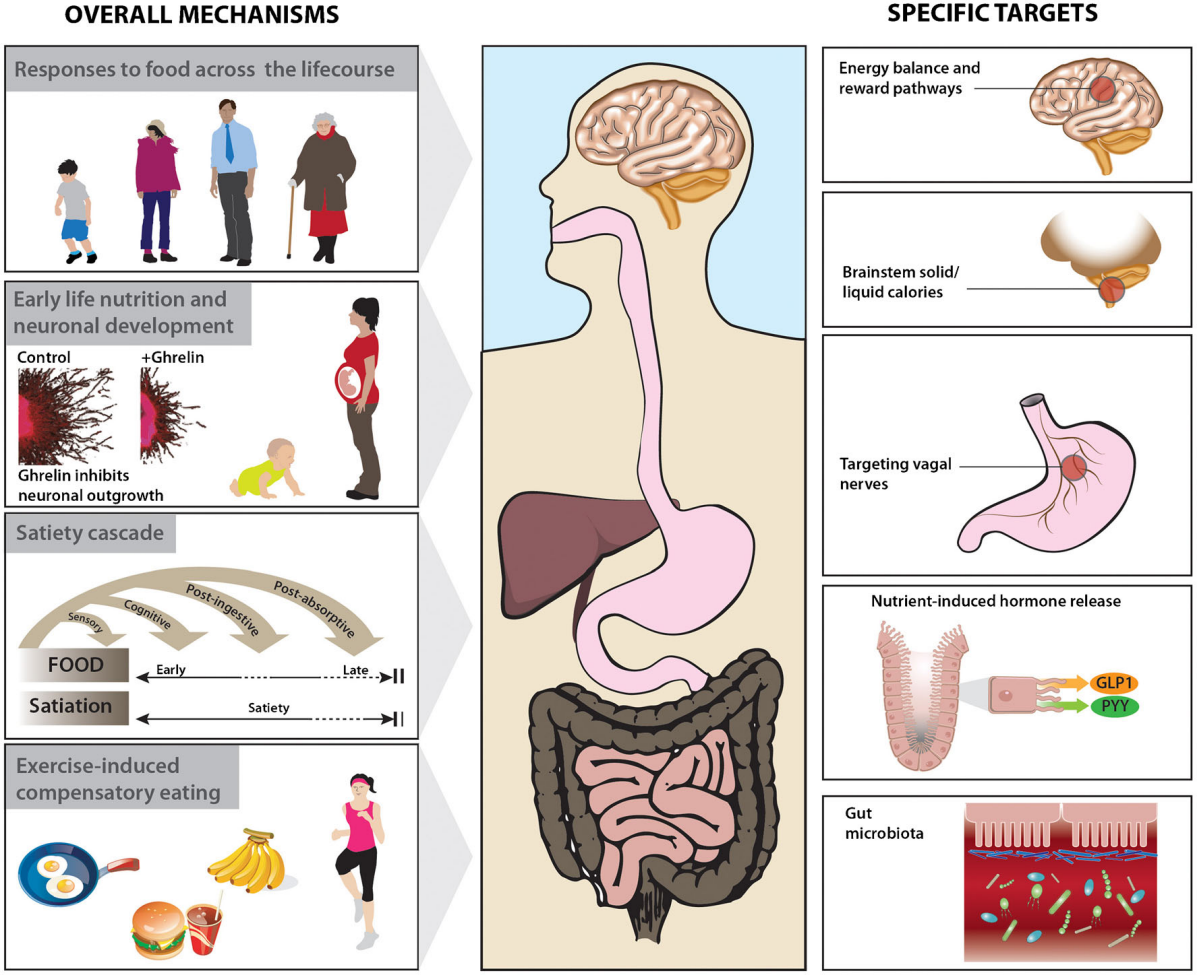
Hunger and satiety: overview of mechanisms and specific targets. Selected aspects of the Full4Health project include responses to food intake across the life course, the impact of early life nutrition on neuronal development and the effect of exercise on feeding behaviour. An integrated physiological system—the ‘food-gut-brain axis’—controls what we choose to eat, when we eat it and the impact on our subsequent behaviour and thus on body weight. Different foci in this axis, as discussed in the text, may provide targets for adapting and exploiting responses to food and could deliver alternative solutions to the problem of overweight and obesity

The satiety cascade (Fig. 1) proposed by Blundell and colleagues more than 20 years ago has been described and embellished subsequently in a number of excellent reviews (e.g. [12, 13]). It is a conceptual framework which combines the physiological events controlling appetite with the simultaneous behaviours and psychological experiences that are integral to the eating process [12]. Hunger is a familiar early ‘signal’ or state leading to the initiation of the eating process, particularly as it relates to meals, whilst the accumulation of signals arising from the act of eating ultimately results in the termination of the eating event. The most commonly perceived hunger signals originate in the stomach where electrical (vagus nerve) signals relate the state of emptiness (or fullness), reinforced by the secretion of the hormone, ghrelin, and by metabolic signals such as blood glucose (hypoglycaemia). *Satiation*, or intra-meal satiation, is the process leading to meal termination and determines meal size. The physiological state at the end of a meal when further eating is inhibited by ‘fullness’ is termed *satiety* [12]. *Satiety*, or between-meal satiety, ends as meal processing and absorptive signals wane and hunger initiates the next period of eating. *Sensory* and *cognitive* processes guide meal anticipation and learned associations with anticipated reward and pleasure, helping to define overall meal quality and quantity. The stomach and intestines provide *post-ingestive* information through the physical signals of stretch/distension as well as osmotic load, providing feedback related to meal quantity. Medium-term satiety is metabolically controlled by gut peptide hormones including GLP-1, cholecystokinin (CCK) and PYY which are released as digesta pass through the gastrointestinal tract and have meal-processing roles in addition to their inhibitory effects on food intake [14]. The *post-absorptive* phase is when long-term satiety is controlled by insulin, glucose and amino acid concentrations in the blood and oxidation of nutrients in the liver. The brain integrates signals from all the processes involved in hedonic and homeostatic appetite control, as well as those concerning sensory and metabolic satiety. It may be possible through food reformulation to produce foods that not only suppress appetite but are also desirable to eat, in order to influence behaviour change and facilitate healthier food choices.

### Current Research Focus—EU Projects

The complexity of the processes involved in feeding behaviour and the contribution of caloric over-consumption to the rising obesity epidemic has driven the need for further research. This has been recognised by the European Union (EU) which has funded two related multidisciplinary Framework Programme 7 (FP7) projects, Full4Health and SAtiety INnovation (SATIN). Full4Health (http://www.full4health.eu/) is investigating the mechanisms controlling hunger and satiety, whilst SATIN (http://www.satin-satiety.eu/) seeks to establish the proof of concept of food reformulation to enhance satiety leading to long-term health benefit.

Full4Health is a multidisciplinary project focusing on the mechanisms controlling hunger, satiety and feeding behaviour, studying the effects of diet, dietary components and food structure (Fig. 1). It includes studies of the gut and the signalling systems (neural, hormonal and metabolic) connecting it to different brain areas. Although not directly an obesity project, its aim is to provide an evidence base on which to build solutions to address over-consumption as part of the burgeoning obesity problem. The mechanisms elucidated could equally be manipulated to address under-consumption of calories (malnutrition) such as that seen in the elderly or clinically compromised, for example in cancer cachexia or following surgery.

A major focus is a human study investigating the effects of dietary intervention on different age, BMI and gender groups, to illustrate the anticipated variations in appetite regulation across the lifespan. Linked to this is the neuropsychology of food choice and reward and how it relates to appetite regulation. In addition to this and other human intervention studies, mechanisms are being investigated using a number of other approaches including cell-based systems and preclinical models, employing cutting-edge technologies such as brain imaging. These techniques are being applied to a number of areas of interest including (i) studies during early life and the impact of nutritional and endocrine manipulations on the development of metabolic disease in later life, (ii) whether the physical form of food (liquid or solid) has different effects on hindbrain processing, and (iii) the role of the vagus nerve in communicating signals from the stomach to the brain (part of the ‘gut-brain axis’).

SATIN has several similar themes to Full4Health and was conceived to address the problem of sustainable weight management through dietary solutions based on functional food products. It is a proof of concept study with the aim of identifying novel food structures that can be incorporated into satiety-enhancing foods which can be tested for their effects on long-term appetite regulation. This ambition reflects the regulatory environment in Europe where the European Food Safety Agency (EFSA) is responsible for overseeing the safety and efficacy of food products across the EU and the claims that food manufacturers can make about their products. In the case of satiety enhancement, the requirements are stringent, and food intake-related or satiety claims for a food will only be approved if they also result in sustained beneficial effects on body weight. The project involves screening novel food structures to identify and characterise satiety-enhancing foods, followed by the testing of lead formulations through satiety and health screens, consumer evaluation and ultimately long-term human volunteer studies. Its aim is to find potential foods which can accelerate within-meal satiation, prolong between-meal satiety, and reduce snacking between meals [15, 16], but importantly, deliver these outcomes as part of a whole diet approach that will be beneficial to long-term health through positive effects on body weight. Such benefits may not necessarily be direct, and products with an approved satiety claim may help consumers to make better food choices, thereby assisting with the maintenance of healthy weight [15, 16, 17, 18, 19, 20, 21, 22, 23].

### Full4Health Research Progress

In this final year of Full4Health, we now report a number of significant developments and scientific advances in our understanding of mechanisms of hunger and satiety and consider how we might utilise these in the battle against obesity (Fig. 1).

As discussed previously, bariatric surgery, although very effective, is generally only deployed in cases of extreme obesity. Full4Health’s Norwegian partner in Trondheim, led by Duan Chen, is investigating less invasive interventions seeking to achieve similar weight loss and clinical outcomes as currently available surgery. They have demonstrated that blocking the gastric vagus nerve input to the brain results in decreased food consumption in a preclinical model (unpublished). The stomach wall is rich in vagus nerve afferents that play various physiological roles in communication between the stomach and the brain (part of the so-called gut-brain axis). Botox (a toxin which blocks release of the neurotransmitter, acetylcholine, from nerve terminals) has been used to block the vagus nerve in preclinical trials in rats. After injecting Botox into the stomach wall of rats that have become obese by feeding on a high fat diet, it was found that the treatment reduced food intake, with resultant reductions in body weight of 25–30 % over a course of 4 weeks [24]. Botox injection also enhanced weight loss (up to 25 %) in obese rats that had previously undergone sleeve gastrectomy surgery [24]. These promising results suggest that this simple treatment has the potential to be refined into a new less invasive therapy for obesity. The vagus nerve is also involved in the control of gastric emptying, but the Trondheim group has found that Botox injection did not delay gastric emptying and did not cause any pathological changes in the stomach [24]. There is a precedent for the use of Botox injection in the clinic—in the treatment of patients with achalasia, a condition where the lower oesophageal sphincter remains closed due to failure of the smooth muscle to relax. So, the technology already exists for Botox to be easily injected through gastroscopy, with the whole procedure requiring the patient to stay in hospital for only a few hours. The promising results generated by Professor Chen’s group have led to initiation of a Phase II clinical trial at St. Olav’s Hospital, Trondheim, Norway. Obese patients have been recruited, and the trial is ongoing. The hope is that this simple procedure can become an effective method for treating obese patients with little risk and significantly lower cost in comparison to obesity surgeries currently used.

It is well-documented that there are strong associations between early life environment and the risk of developing metabolic disease, including obesity and diabetes, in later life [2]. Full4Health partner at the University of Lille, led by Sebastien Bouret, is investigating early life effects of the stomach hormone, ghrelin. Foetal nutrition was first recognised to affect long-term metabolic health following study of individuals born in the Dutch Hunger Winter at the end of the Second World War [25]. The sudden onset of the famine and its short duration of only 5 months provided a remarkable opportunity to compare its effects with the periods before and after it. Studies showed that women exposed to the famine during early pregnancy gave birth to normal weight babies. However, these offspring had a higher incidence of obesity in later life than those born before or after the famine and also than those lower birth weight babies who were exposed to the famine in mid-late gestation [25]. The lower birth weight babies maintained their lower weight throughout life and had lower obesity rates. Although the observations made from this study form the basis of key information on foetal programming, it is clear that preclinical studies are necessary to dissect out the processes and key factors in operation, generating knowledge that cannot be gleaned from ‘natural experiments’ in man.

Bouret’s group in Lille has inhibited ghrelin action during the early postnatal period in mice and revealed an important role for this hormone in the development of hypothalamic neural circuits; ghrelin blockade enhanced arcuate nucleus neural projections. They also showed that *increasing* ghrelin levels during the postnatal period, and disrupting the normal development of arcuate nucleus neural projections, resulted in lifelong metabolic disturbances that were apparent in older mice, including increases in body weight, higher circulating leptin and insulin levels and increased glycaemia [26••]. This is an important development in our understanding of the diverse roles of gut-brain peptide hormones, juxtaposing ghrelin with the adipose tissue hormone, leptin, which is a stimulant of neurite growth—thus *inhibition* of the action of leptin similarly inhibits neural development [27]. It has been known for some time that leptin, whilst having a key role in energy balance and signalling of body fat stores in post-weaning life in the rodent, also has a neurodevelopmental role during neonatal life, as exemplified by the so-called ‘leptin surge’. The mechanistic studies conducted as part of Full4Health have added an additional level of complexity to our understanding of the regulatory processes through which the mapping of the developing hypothalamic circuitry is determined, with potential consequences for lifelong health. The Lille group has also shown that overnutrition by litter size manipulation during the early postnatal period reduces ghrelin levels, leading to metabolic effects which could not be reversed by injecting ghrelin. This disturbance of the ghrelin system, which may manifest itself as ‘resistance’, may be important in explaining the metabolic defects in postnatally overnourished mice [28].

The knowledge that leptin and ghrelin may have counter-regulatory actions during the postnatal period and combine to shape the correct development of brain feeding circuits is an important advance. This will be valuable in designing interventions, perhaps applicable to maternal/postnatal nutrition in humans, to address metabolic disease, including the rising obesity problem.

The enteroendocrine system distributed along the intestinal tract secretes a number of regulatory peptides with satiety properties. The mechanisms underlying the beneficial effects of bariatric surgery in countering both diabetes and obesity remain to be fully elucidated but are believed to reflect, at least in part, the activity of nutrient-released gastrointestinal and pancreatic peptides. Harnessing the potential of this secretory capability is a strategy being pursued within the Full4Health project, with the broad aim of mimicking bariatric surgery through nutrient targeting. GLP-1 is produced by L cells in the distal ileum, stimulated by ingested nutrients. Ingested protein stimulates the release of GLP-1, and it is also established that individual amino acids, such as glutamine when administered orally, can elevate levels of GLP-1 in lean, obese and type 2 diabetic individuals [29, 30]. Full4Health researchers at the University of Cambridge found that oligopeptides triggered release of GLP-1 via two signalling pathways in L cells in vitro. The components of these pathways are highly expressed and are relatively specific for L cells, suggesting that they may provide a route to increasing endogenous GLP-1 secretion, with all the expected benefits which may result [31••]. This work has been used to inform research using a preclinical perfused isolated intestine model elsewhere in the Full4Health consortium [32]. Such synergistic activity illustrates clearly how fundamental mechanistic studies on GI hormones may provide a novel way to harness their satiety-enhancing activity and deliver solutions to the challenge of escalating obesity levels.

Full4Health has grown the evidence base of mechanisms of satiety and feeding behaviour, which may in the future provide support for the majority of the population who are overweight, and thereby slow the progression to obesity. However, potential mechanism-based interventions are most likely to deliver success if combined with lifestyle modification, such as exercise. The University of Leeds is a Full4Health partner investigating the effects of exercise on appetite behaviour. Previous studies had supported the idea that exercise produces less weight loss than anticipated and that there are gender differences, with females losing less weight than males [33, 34]. However, it is important for such studies to quantify energy intake and expenditure. Graham Finlayson and colleagues in Leeds measured energy expenditure during supervised exercise, comparing changes in body composition in studies of males and premenopausal females. They found no effect of gender on changes in body fat content during the supervised exercise programme [35]. They also noted that the exercise schedule increased fasting hunger but that this did not result in higher food intake. A suggested explanation is that even though hunger was increased, postprandial satiety also increased [36]. This may be explained by suggestions that long-term exercise increases the levels of the satiety hormones, GLP-1 and PYY [35].

### SATIN Research Progress

Progress in this project is discussed in another article, also in this issue.

## Conclusion

Strategies to deal with the rise of obesity have had mixed results. Bariatric surgery is the most successful of currently available interventions but is only really applicable to the more extreme clinical cases. Pharmacological therapy has been beset by problems of adverse reaction. A larger proportion of the population are overweight rather than obese, but many are tracking towards obesity, due to incremental increases in weight over many years. We need solutions to stem this gradual upward trajectory at as early a stage as possible. It is accepted that calorie restriction in the form of dieting generally delivers rather limited success in weight management, an outcome which probably reflects the fact that this strategy is being countered by the body’s natural physiological response to negative energy balance. Extensive research efforts over the last 30 or so years have, however, revealed much of the molecular and neuroanatomical detail of the control of energy balance, involving the GI tract, gut peptides, peripheral nerves, and neuroendocrine and reward systems in the brain, and how food interacts with these systems and processes. Perhaps it is now time for a new approach to try to address the problems of over- and also under-consumption of calories by using the natural properties of food, such as differential induction of satiation and satiety, to enable individuals to control hunger (the biggest reason for dietary failure) and make better food choices. Full4Health has delivered mechanistic information regarding hunger and satiety to inform this process and SATIN is taking this to the next step by testing the concept of ‘designer’ foods which aim to change the way we eat. It should be borne in mind however that this approach will be most likely to yield success when combined with lifestyle modifications such as exercise.
